# Supplementary material for: Phage Display Revealed the Complex Structure of the Epitope of the Monoclonal Antibody 10H10
Source: Int J Mol Sci. 2024 Sep 25;25(19):10311. doi: 10.3390/ijms251910311 (PMC11476565; doi:10.3390/ijms251910311)
Supplement: Supplementary file 1 [file ijms-25-10311-s001.zip › Supplementary figures.pdf]

Supplementary materials:

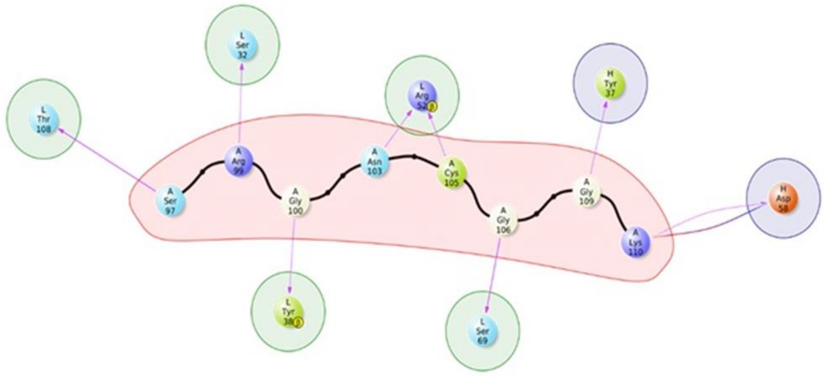

(a)

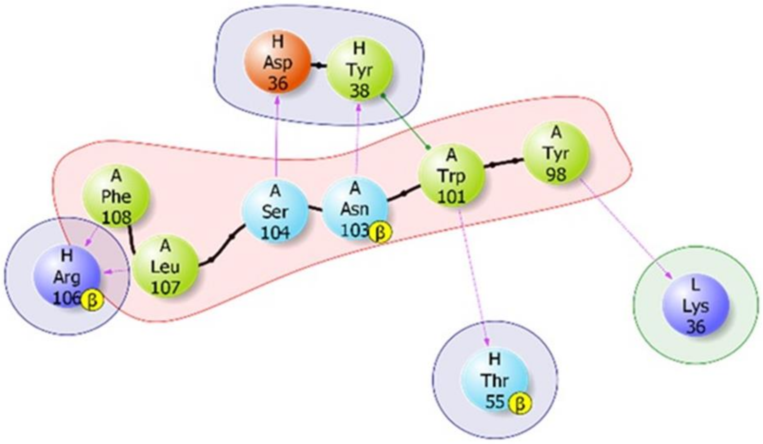

(b)

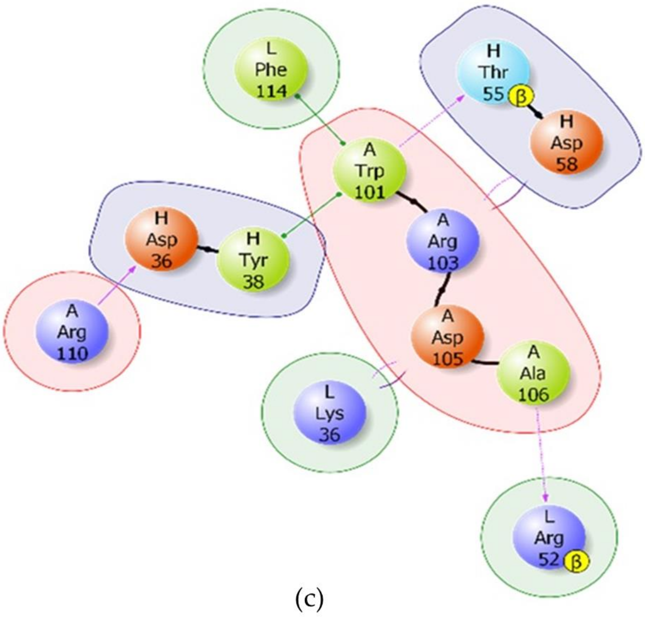

(c)

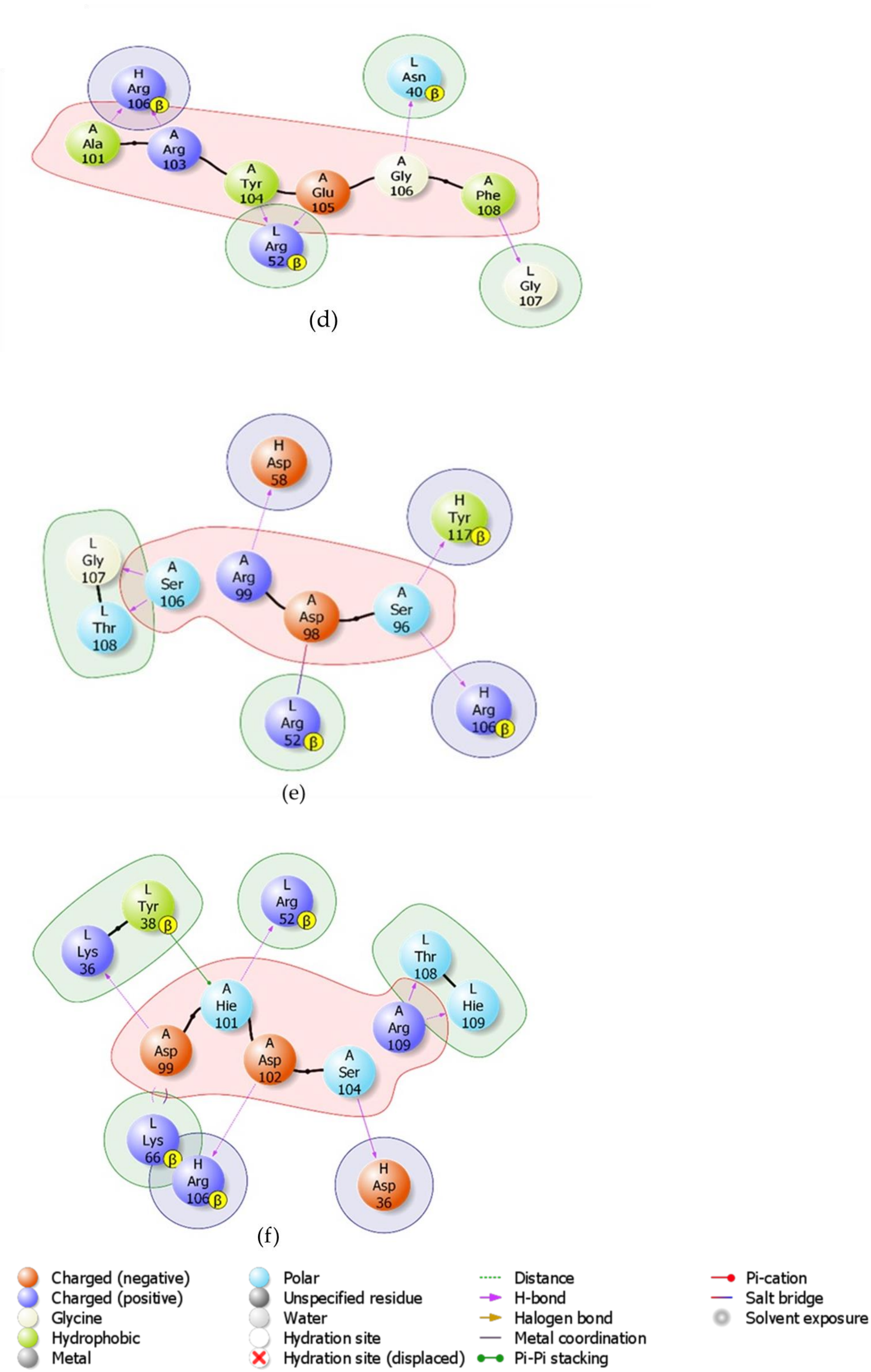

Figure S1 Molecular docking results

A – The diagrams of interaction between a.a. of 10H10 with a.a. of peptides FL; B – The diagrams of interaction between a.a. of 10H10 with a.a. of peptides 1; C – The diagrams of interaction between a.a. of 10H10 with a.a. of peptides 2; D – The diagrams of interaction between a.a. of 10H10 with a.a. of peptides 3; E – The diagrams of interaction between a.a. of

10H10 with a.a. of peptides 4; F – The diagrams of interaction between a.a. of 10H10 with a.a. of peptides 5

Supplementary materials:

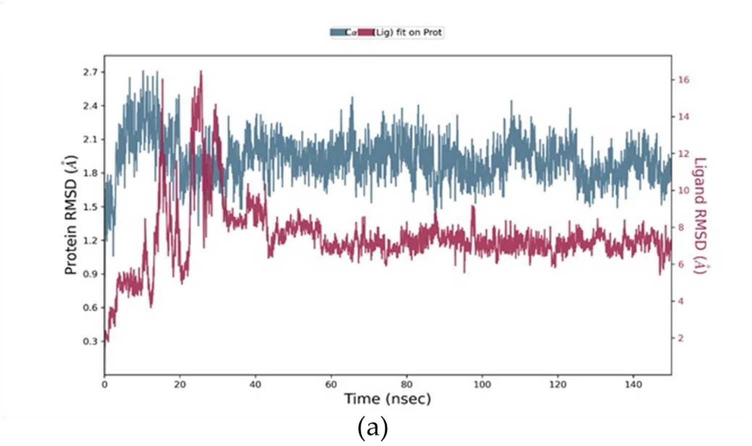

(a)

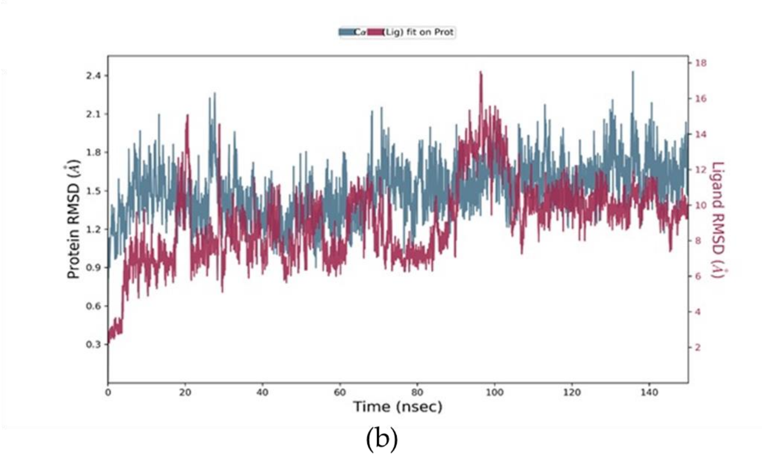

(b)

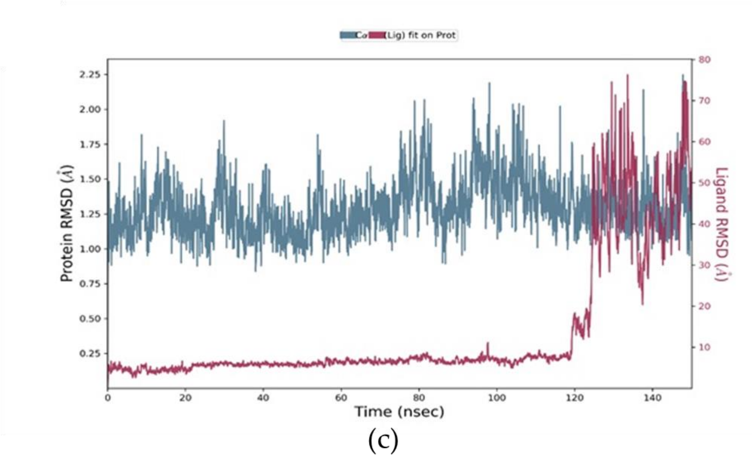

(c)

Figure S2 Molecular dynamics simulations

A – RMSD of pep 1-10H10 complex; B – RMSD of pep 2-10H10 complex; C – RMSD of pep 5-10H10 complex
